# The Neurohormonal Overactivity Syndrome in Heart Failure

**DOI:** 10.3390/life13010250

**Published:** 2023-01-16

**Authors:** Andrew Xanthopoulos, John Skoularigis, Filippos Triposkiadis

**Affiliations:** Department of Cardiology, University General Hospital of Larissa, 41110 Larissa, Greece

**Keywords:** neurohormonal, overactivity, syndrome, heart failure, sodium glucose co-transporter 2 inhibitors

## Abstract

Heart failure (HF) is categorized arbitrarily based on the left ventricular ejection fraction (LVEF) in HF with reduced (HFrEF; LVEF < 40%), mildly reduced (HFmrEF; LVEF 40–49%), or preserved ejection fraction (HFpEF; LVEF ≥ 50%). In this opinion paper, based on (patho)physiological considerations, we contend that the neurohormonal overactivity syndrome (NOHS), which is present in all symptomatic HF patients irrespective of their LVEF, not only contributes to the development of signs and symptoms but it is also a major determinant of patients’ outcomes. In this regard, NHOS is the only currently available treatment target in HF and should be combatted in most patients with the combined use of diuretics and neurohormonal inhibitors (β-blockers, angiotensin receptor-neprilysin inhibitor/angiotensin-converting enzyme inhibitors/angiotensin receptor blockers, mineralocorticoid antagonists, and sodium-glucose co-transporter 2 inhibitors). Unfortunately, despite the advances in therapeutics, HF mortality remains high. Probably machine learning approaches could better assess the multiple and higher-dimension interactions leading to the HF syndrome and define clusters of HF treatment efficacy.

## 1. Introduction

**Figure 1:**
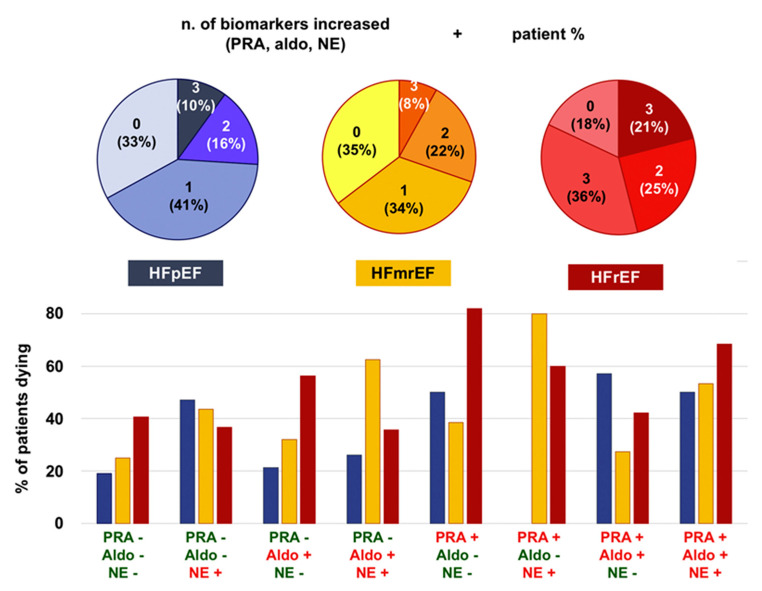
Sympathetic and renin-angiotensin-aldosterone system activation across ejection fraction categories and their prognostic impact. Aldo, aldosterone; HFmrEF, heart failure with mildly reduced ejection fraction; HFpEF, heart failure with preserved ejection fraction; HFrEF, heart failure with reduced ejection fraction; NE, norepinephrine NT-proBNP, N-terminal fraction of pro-B-type natriuretic peptide PRA, plasma renin activity. Adapted with permission from Ref. [8]. © 2019 Elsevier B.V.

Heart failure (HF) is a clinical syndrome related to high morbidity and mortality [1]. According to the traditional view, HF is categorized based on the left ventricular ejection fraction (LVEF) in HF with reduced (HFrEF; LVEF < 40%), mid-range (HFmrEF; LVEF 40–49%), or preserved ejection fraction (HFpEF; LVEF ≥ 50%). Nevertheless, LVEF categorization has several limitations (i.e., imprecise physiological implications, substantial intra- and inter-observer variability between LVEF measurements, arbitrary LVEF cut-offs, LVEF transitions) and has been challenged (i.e., epidemiological, clinical, pathophysiological, and therapeutic features are common across the HF spectrum) [2,3,4,5,6]. In this regard, the neurohormonal overactivity syndrome (NHOS), which is present in all symptomatic HF patients, irrespective of the LVEF, contributes to the development of signs and symptoms (please see Pathophysiology section), is a major determinant of outcomes [7,8] (Figure 1) and is the only currently available treatment target to reduce rehospitalizations and prolong survival [5].

## 2. Pathophysiology

Risk factors (hypertension, coronary artery disease, obesity, and others) always precede the development of HF and are associated with a high HF incidence, whereas comorbidities (atrial fibrillation, diabetes mellitus, anemia, chronic kidney disease, depression, pulmonary diseases, sleep-disordered breathing, and others) may precede or develop after HF and usually co-exist with HF in groups of two or more (multi-morbidity) [9]. The complex interaction between risk factors, comorbidities, and disease modifiers (sex, genes, and others) may lead to cardiac damage (myocardial dysfunction) and HF [10].

**Figure 2:**
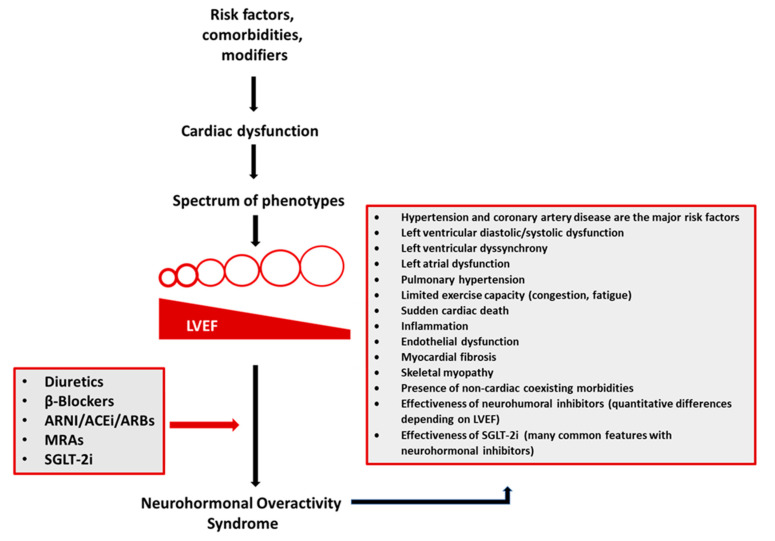
A complex interplay between risk factors (coronary artery disease, hypertension, obesity, and others), comorbidities (atrial fibrillation, diabetes mellitus, chronic kidney disease, depression, pulmonary diseases, sleep-disordered breathing, anemia, and others), and disease modifiers (sex, genes, and others) leads to cardiac damage manifested by a spectrum of phenotypes. Diverse heart failure (HF) phenotypes converge to the neurohormonal overactivity syndrome (NOHS). Treatment with diuretics and neurohormonal inhibitors (β-blockers, angiotensin receptor-neprilysin inhibitor (ARNI)/angiotensin-converting enzyme inhibitors (ACEi)/angiotensin receptor blockers (ARBs), mineralocorticoid antagonists (MRAs), and sodium-glucose co-transporter 2 inhibitors (SGLT-2i)) is the only currently available treatment to combat NHOS and, therefore, decrease morbidity and mortality in most HF patients.

Traditionally, the principal hemodynamic mechanisms following cardiac damage have included low cardiac output at rest or even at exercise (“forward failure”) as well as elevated (left and/or right) cardiac filling pressures (“backward failure”) [11]. Rarely cardiac output is increased in HF due to vasodilation and decreased peripheral resistance (high high-output HF) [12]. The studies on patients with low- and high-output HF, support the idea that preservation or maintenance of the arterial blood pressure is the main trigger for the development of neurohormonal overactivity (activation of the sympathetic nervous system (SNS) and the renin-angiotensin aldosterone system (RAAS)) [13,14]) signaling to the kidney to retain sodium and water which seen in all forms of HF [15]. However prolonged neurohormonal overactivity results in endothelial dysfunction, myocardial fibrosis, skeletal myopathy, and inflammation [16]. The concentration of circulating proinflammatory cytokines is increased, presumably due to endotoxin-induced immune activation resulting from bowel oedema, myocardial production due to haemodynamic overload, and peripheral extramyocardial production due to tissue hypoperfusion and hypoxia [17] and may stimulate the production of reactive oxygen species (ROS) via induction of the NADPH oxidase [18]. The above result in neurohormonal overactivity syndrome (NHOS), which is characterized by exercise intolerance, limited by dyspnea and fatigue (Figure 2). Congestion, the cardinal derangement underlying NHOS, is due to renal retention of sodium and water and leads to intravascular and interstitial fluid volume expansion and redistribution [19]. Although initial sympathetic-driven vasoconstriction supports organ perfusion pressure in the short term, a more progressive aggregation of interstitial compartment fluid also arises which supports a compensatory expansion of intravascular plasma volume. A fast component, which is noticed shortly prior to decompensation, is triggered by the sympathetic nervous system overactivity and leads to an intercompartmental fluid shift into the central circulation with an upcoming increase in central filling pressures [20].

The detrimental consequences of the prolonged activation of the RAAS and SNS are partly counteracted by the activation of neurohumoral factors such as natriuretic peptides (atrial (ANP), brain (BNP), and C-type natriuretic peptides (CNP)), which exhibit cardioprotective effects (i.e., diuretic, natriuretic, and vasodilatory actions) [21,22]. Unfortunately, although patients with chronic HF have an increase in ANP and BNP production, the proportion of inactive molecules is increased along with an increase in natriuretic peptide-degrading enzymes and the receptor-mediated clearance of natriuretic peptides [23].

The advanced stage of NHOS is characterized by the development of multiorgan dysfunction due to the development of an abnormal bidirectional relationship between the heart and several organs such as the brain (cardiocerebral syndrome) [24], the kidney (cardiorenal syndrome) [25] or the liver (cardiohepatic syndrome) [26] and leading to a vicious cycle.

Finally, sudden cardiac death (SCD) is a cardinal manifestation of the NHOS syndrome [27]. Heart disease disposes patients towards malignant ventricular arrhythmias by causing neural remodeling at the level of the myocardium, the intrinsic cardiac ganglia, spinal cord, extracardiac intrathoracic sympathetic ganglia, extrathoracic ganglia, and the brainstem, as well as the higher centers and the cortex [28]. Abnormal metabolism and oxidative stress in the cardiomyocytes and noncardiac myocytes tissues also may lead to ventricular arrhythmias and SCD [29]. Treatment with neurohumoral inhibitors dramatically reduces the risk of SCD emphasizing the tight link between neurohormonal overactivity and SCD [30]. In particular, an analysis including 40,195 HF patients from 12 clinical trials conducted over a period of 19 years, demonstrated that the use of angiotensin-converting–enzyme inhibitors, angiotensin-receptor blockers, angiotensin receptor-neprilysin inhibitors, beta-blockers, and mineralocorticoid-receptor antagonists was associated with 44% lower risk of SCD [30]. Several recent meta-analyses of randomized controlled trials on sodium-glucose co-transporter 2 inhibitors (SGLT-2i) revealed a trend towards reduced risk of ventricular tachycardia or fibrillation and sudden cardiac death in patients with type 2 diabetes mellitus as well as patients with HF [31,32,33,34]. Nevertheless, in the majority of these meta-analyses, statistical significance was not reached, presumably due to the low number of events [35].

## 3. Clinical Implications

NHOS is the only currently available treatment target in HF and should be combatted in most patients with the combined use of diuretics and neurohormonal inhibitors (β-blockers/angiotensin-converting enzyme inhibitors/angiotensin receptor blockers, mineralocorticoid antagonists, and SGLT-2i) [5,36,37,38,39]. The novel SGLT-2i block glucose reabsorption in the renal proximal tubule, a mechanism independent of insulin that plays an important role in glycemic regulation in diabetes [40]. Aside from the target effects of SGLT-2i, a wide variety of favorable effects is described for the kidney and the heart, despite the fact that cardiac tissue does not express SGLT-2 channels. SGLT-2i decrease the hazard of death from cardiac or vascular causes and hospitalization for HF in patients with HF. Initially, these medicines were thought to exert actions similar to diuretics or hemodynamically active drugs, but they do not rapidly decrease natriuretic peptides or cardiac filling pressures, and they demonstrate minimal early benefit on symptoms, exercise tolerance, or quality of life. Clinically, the profile of SGLT-2i looks similar to that of neurohormonal inhibitors, whose favorable effects are observed gradually during sustained therapy. Indeed, in experimental in vivo animal models, SGLT-2i produce a distinctive pattern of cellular effects (attenuation of proinflammatory pathways, amelioration of oxidative stress, mitigation of mitochondrial dysfunction, and a reduction in myocardial fibrosis), which is similar to that of neurohormonal inhibitors [41]. At the molecular level, SGLT-2i lead to transcriptional reprogramming of cardiomyocytes that closely resembles that observed during nutrient deprivation. This alteration in signaling triggers the housekeeping pathway of autophagy, which clears the cytosol of threatening cytosolic constituents that cause cellular stress, thereby decreasing the risk of cardiomyopathy development [42]. Remarkably, similar alterations in cellular signaling and autophagic flux have also been observed in neurohormonal inhibitors.

## 4. Conclusions

Most symptomatic HF phenotypes converge on the NHOS which is an important outcome predictor and one of the rare rewarding treatment targets in HF. As the NHOS is present throughout the HF spectrum of phenotypes, the LVEF-based HF classification and treatment guidelines should be abandoned as this strategy has deprived HF patients of years of potentially lifesaving treatments. As HF mortality remains high despite the advances in therapeutics, machine learning approaches could better assess the multiple and higher-dimension interactions leading to HF syndrome and define clusters of HF treatment efficacy. This strategy has already been successfully used in HF both for more accurate phenotyping [43] and the implementation of individualized treatment [44].

## Data Availability

Not applicable.
